# Supplementary material for: Three-Dimensional Osteogenic Differentiation of Bone Marrow Mesenchymal Stem Cells Promotes Matrix Metallopeptidase 13 (MMP13) Expression in Type I Collagen Hydrogels
Source: Int J Mol Sci. 2021 Dec 18;22(24):13594. doi: 10.3390/ijms222413594 (PMC8706974; doi:10.3390/ijms222413594)
Supplement: Supplementary file 1 [file ijms-22-13594-s001.zip › ijms-1509202-supplementary.pdf]

International Journal of *Molecular Sciences*

MDPI

Supplementary Material

Three-Dimensional Osteogenic Differentiation of Bone Marrow Mesenchymal Stem Cells Promotes Matrix Metallopeptidase 13 (MMP13) Expression in Type I Collagen Hydrogels

Luis Oliveros Anerillas [1], Paul J. Kingham [1], Mikko J. Lammi [1], Mikael Wiberg [1,2] and Peyman Kelk [1,*]

[1] Department of Integrative Medical Biology, Umeå University, 901 87 Umeå, Sweden; luis.oliveros@umu.se (L.O.A.); paul.kingham@umu.se (P.J.K.); mikko.lammi@umu.se (M.J.L.); mikael.pj.wiberg@umu.se (M.W.)
[2] Department of Surgical & Perioperative Sciences, Section for Hand and Plastic Surgery, Umeå University, 901 87 Umeå, Sweden
* Correspondence: peyman.kelk@umu.se

Table S1: Gene sequences used by NanoString®´s nCounter System

Gene name	Accession	Gene Sequence
**ABCF1**	NM_001090.2	GATGTCCTCCCGCCAAGCCATGTTAGAAAATGCATCTGACATCAAGCTGGAGAAG TTCAGCATCTCCGCTCATGGCAAGGAGCTGTTCGTCAATGCAGAC
**ALPL**	NM_000478.3	CAGGGTAGATTTCTCTTGGGCAGGCAGAGAGTACAGACTGCAGACATTCTCAAAG CCTCTTATTTTTCTAGCGAACGTATTTCTCCAGACCCAGAGGCCC
**BGLAP**	NM_199173.4	AGCAGCCCAGCGCAGCCACCGAGACACCATGAGAGCCCTCACACTCCTCGCCCTA TTGGCCCTGGCCGCACTTTGCATCGCTGGCCAGGCAGGTGCGAAG
**BMP1**	NM_001199.1	GCTTCTTTGCAGTCTACGAAGCCATCTGCGGGGGTGATGTGAAAAAGGACTATGGC CACATTCAATCGCCCAACTACCCAGACGATTACCGGCCCAGCAA
**BMP2**	NM_001200.2	TTAGGATAAGCAGGTCTTTGCACCAAGATGAACACAGCTGGTCACAGATAAGGCC ATTGCTAGTAACTTTTGGCCATGATGGAAAAGGGCATCCTCTCCA
**BMP3**	NM_001201.1	GAGCCAGAAAGTGTGGTATCAAGCTTACAGGGACACCGGAATTTTCCCACTGGAA CTGTTCCCAAATGGGATAGCCACATCAGAGCTGCCCTTTCCATTG
**BMP4**	NM_001202.3	GACTACATGCGGGATCTTTACCGGCTTCAGTCTGGGGAGGAGGAGGAAGAGCAGA TCCACAGCACTGGTCTTGAGTATCCTGAGCGCCCGGCCAGCCGGG
**BMP5**	NM_021073.2	GACCATTTTCACCTGGAAAACAAGCGTCCTCTGCACCTCTCTTTATGCTGGATCTCT ACAATGCCATGACCAATGAAGAAAATCCTGAAGAGTCGGAGTA
**BMP6**	NM_001718.2	CCTGTTTTTGTTGGACACCCGTGTAGTATGGGCCTCAGAAGAAGGCTGGCTGGAAT TTGACATCACGGCCACTAGCAATCTGTGGGTTGTGACTCCACAG
**BMP7**	NM_001719.1	GCTTCGTCAACCTCGTGGAACATGACAAGGAATTCTTCCACCCACGCTACCACCAT CGAGAGTTCCGGTTTGATCTTTCCAAGATCCCAGAAGGGGAAGC
**BMPR1A**	NM_004329.2	AAATGAAGTTGATGTGCCCTTGAATACCAGGGTGGGCACCAAACGCTACATGGCT CCCGAAGTGCTGGACGAAAGCCTGAACAAAAACCACTTCCAGCCC
**BMPR1B**	NM_001203.1	GTTTCACGATGATAGAAGAGGATGACTCTGGGTTGCCTGTGGTCACTTCTGGTTGC CTAGGACTAGAAGGCTCAGATTTTCAGTGTCGGGACACTCCCAT
**CALCR**	NM_001742.2	TTACATCTGCCATCAGGAGCTGAGGAATGAACCAGCCAACAACCAAGGCGAGGA GAGTGCTGAGATCATCCCTTTGAATATCATAGAGCAAGAGTCATCT
**COL10A1**	NM_000493.3	AACTTGGTTCATGGAGTGTTTTACGCTGAACGATACCAAATGCCCACAGGCATAAA AGGCCCACTACCCAACACCAAGACACAGTTCTTCATTCCCTACA
**COL11A1**	NM_001190709.1	GTAAAGGGAGCAGATGGTGTCAGAGGTCTCAAGGGATCTAAAGGTGAAAAGGGT GAAGATGGTTTTCCAGGATTCAAAGGTGACATGGGTCTAAAAGGTG
**COL12A1**	NM_004370.5	GGTACCAATCCCCGGGAATACCAATTATGCCATTCTTAGGAATCTGCAGCCAGATA CCTCATACACTGTGACTGTAGTTCCCGTTTATACTGAAGGTGAT
**COL14A1**	NM_021110.1	CTTTAAGTCCACCAAGAAACCTGAGAATCTCCAATGTTGGCTCTAACAGTGCTCGA TTAACCTGGGACCCAACTTCAAGACAGATCAATGGTTATCGAAT
**COL15A1**	NM_001855.2	CCCAATACCCGTCCGACCACACTGCAAAATGCCAGTTGATACTGCTCATCCTGGGA GTCCAGAGCTCATCACTTTTCACGGTGTTAAAGGAGAGAAAGGA
**COL16A1**	NM_001856.3	CCCTGCTTGTCCTGCAGCTCGGTTGTAGGGGCCCAGCATCTTGTGTCCTCCACAGG GGCCAGTGGAGATGTGGGTTCCCCTGGCTTTGGTCTGCCTGGCC
**COL17A1**	NM_000494.3	TGACACTTGATGGGGGTGTGTTCTGGTTACTGTTCTAAGGCTGTGCCATCAGCTCCT TCCTCCCCTGTTCATTCTGCATTCTCTAGTCAGTTGGCTAAGA
**COL18A1**	NM_030582.3	CACCTGCCTCAGGACTGCGACGAAACCGGTGGGGCTGGTTCTGTAATTGTGTGTGA TGTGAAGCCAATTCAGACAGGCAAATAAAAGTGACCTTTTACAC
**COL19A1**	NM_001858.5	AGACTCACTGGCCCTTGGAAACTTTGGCTTTGGATGTCAATATTTCTGCTTCCTGCT TCCACTTCCGTGACCGTTAGGGACAAGACAGAAGAGTCATGCC
**COL1A1**	NM_000088.3	CAGAAACATCGGATTTGGGGAACGCGTGTCAATCCCTTGTGCCGCAGGGCTGGGC GGGAGAGACTGTTCTGTTCCTTGTGTAACTGTGTTGCTGAAAGAC
**COL1A2**	NM_000089.3	CCAATGGATTTGCTGGTCCTGCTGGTGCTGCTGGTCAACCTGGTGCTAAAGGAGAA AGAGGAGCCAAAGGGCCTAAGGGTGAAAACGGTGTTGTTGGTCC

**COL2A1**	NM_001844.4	GACCTGATGTCCATTCATCCCACCCTCTCACAGTTCGGACTTTTCTCCCCTCTCTTTC<br>TAAGAGACCTGAACTGGGCAGACTGCAAAATAAAATCTCGGT
**COL3A1**	NM_000090.3	TTGGCACAACAGGAAGCTGTTGAAGGAGGATGTTCCCATCTTGGTCAGTCCTATGC<br>GGATAGAGATGTCTGGAAGCCAGAACCATGCCAAATATGTGTCT
**COL4A3**	NM_000091.4	CTGGAAGTGAGGGAGTCAAGGGCAACAGGGGTTTCCCTGGGTTAATGGGTGAAGA<br>TGGCATTAAGGGACAGAAAGGGGACATTGGCCCTCCAGGATTTCG
**COL4A4**	NM_000092.4	TATATGGGAGTGGAAAGAAATACATTGGTCCTTGTGGAGGAAGAGATTGCTCTGTT<br>TGCCACTGTGTTCCTGAAAAGGGGTCTCGGGGTCCACCAGGACC
**COL4A5**	NM_000495.4	GTGACACTTGCTTCAACTGCATTGGAACTGGTATTTCAGGGCCTCCAGGTCAACCT<br>GGTTTGCCAGGTCTCCCAGGTCCTCCAGGATCTCTTGGTTTCCC
**COL5A1**	NM_000093.3	AGTGGCACAGAATTGCTCTCAGCGTCCACAAGAAAAATGTCACCTTGATCCTCGA<br>CTGTAAAAAGAAGACCACCAAATTCCTCGACCGCAGCGACCACCC
**COL7A1**	NM_000094.2	GCTCTGGGGGTGATGTGATCCGCGCCATCCGTGAGCTTAGCTACAAGGGGGGCAA<br>CACTCGCACAGGGGCTGCAATTCTCCATGTGGCTGACCATGTCTT
**COL9A2**	NM_001852.3	ATTCGGCTGTTACCAAAACAAACATCTTAATCTGCACCTTTCTCCACTGGCCATCTT<br>GTCCTTGGGTCAGTGGGACATGGGCACCTCGGGAGGCCCGGGC
**DSPP**	NM_014208.3	GGAAATACTGATAAGAATACCCAAAATGGGGATGTTGGCGATGCAGGTCACAATG<br>AGGATGTCGCTGTTGTCCAAGAAGATGGACCTCAAGTAGCTGGAA
**FGF1**	NM_033137.1	AAATGAGGAATGTTTGTTCCTGGAAAGGCTGGAGGAGAACCATTACAACACCTAT<br>ATATCCAAGAAGCATGCAGAGAAGAATTGGTTTGTTGGCCTCAAG
**FGF2**	NM_002006.4	GTCCGGGAGAAGAGCGACCCTCACATCAAGCTACAACTTCAAGCAGAAGAGAGA<br>GGAGTTGTGTCTATCAAAGGAGTGTGTGCTAACCGTTACCTGGCTA
**FGF3**	NM_005247.2	CTGGAGAACAGCGCCTACAGTATTTTGGAGATAACGGCAGTGGAGGTGGGCATTG<br>TGGCCATCAGGGGTCTCTTCTCCGGGCGGTACCTGGCCATGAACA
**FGFR1**	NM_015850.3	CAGTGACCCGCAGCCGCACATCCAGTGGCTAAAGCACATCGAGGTGAATGGGAG<br>CAAGATTGGCCCAGACAACCTGCCTTATGTCCAGATCTTGAAGACT
**FGFR2**	NM_000141.4	AAAGATGATGCCACAGAGAAAGACCTTTCTGATCTGGTGTCAGAGATGGAGATGA<br>TGAAGATGATTGGGAAACACAAGAATATCATAAATCTTCTTGGAG
**FGFR3**	NM_022965.2	GGACCTGTATATTTGTAAAGCTATTTATGGGCCCCTGGCACTCTTGTTCCCACACCC<br>CAACACTTCCAGCATTTAGCTGGCCACATGGCGGAGAGTTTTA
**FLT1**	NM_002019.4	AAGAAATGGCAAACAATTCTGCAGTACTTTAACCTTGAACACAGCTCAAGCAAAC<br>CACACTGGCTTCTACAGCTGCAAATATCTAGCTGTACCTACTTCA
**GDF10**	NM_004962.2	ATCTCACCGAAATCTTTTGATGCCTACTACTGCGCGGGAGCATGTGAGTTCCCCAT<br>GCCTAAGATCGTTCGTCCATCCAACCATGCCACCATCCAGAGCA
**GUSB**	NM_000181.3	CCGATTTCATGACTGAACAGTCACCGACGAGAGTGCTGGGGAATAAAAAGGGGAT<br>CTTCACTCGGCAGAGACAACCAAAAAGTGCAGCGTTCCTTTTGCG
**HPRT1**	NM_000194.1	TGTGATGAAGGAGATGGGAGGCCATCACATTGTAGCCCTCTGTGTGCTCAAGGGG<br>GGCTATAAATTCTTTGCTGACCTGCTGGATTACATCAAAGCACTG
**IBSP**	NM_004967.3	AGGGGGAGTACGAATACACGGGCGCCAATGAATACGACAATGGATATGAAATCT<br>ATGAAAGTGAGAACGGGGAACCTCGTGGGGACAATTACCGAGCCTA
**LDHA**	NM_001165414.1	AACTTCCTGGCTCCTTCACTGAACATGCCTAGTCCAACATTTTTTCCCAGTGAGTCA<br>CATCCTGGGATCCAGTGTATAAATCCAATATCATGTCTTGTGC
**MMP13**	NM_002427.2	CTGGCGCCTGCATCCTCAGCAGGTTGATGCGGAGCTGTTTTTAACGAAATCATTTTG<br>GCCAGAACTTCCCAACCGTATTGATGCTGCATATGAGCACCCT
**MMP2**	NM_004530.2	CCCGGAGGGGCCTGGCAGCCGTGCCTTCAGCTCTACAGCTAATCAGCATTCTCACT<br>CCTACCTGGTAATTTAAGATTCCAGAGAGTGGCTCCTCCCGGTG
**MMP8**	NM_002424.2	CTCTCTCCTAGAGTCCAAACCCAAATGGGCCAGTTGGATCTGATGTTCGTCAGTTCT<br>TTACTTCTATTTCCTGGGGTACTCAGGAGGGCACACACTATAG
**NANOG**	NM_024865.2	CTACTCCATGAACATGCAACCTGAAGACGTGTGAAGATGAGTGAAACTGATATTA<br>CTCAATTTCAGTCTGGACACTGGCTGAATCCTTCCTCTCCCCTCC
**PDGFA**	NM_002607.5	CGTTTGTGGCTGAGTGACAACTTGTTCCCCGCAGTGCACACCTAGAATGCTGTGTT<br>CCCACGCGGCACGTGAGATGCATTGCCGCTTCTGTCTGTGTTGT

**PHEX**	NM_000444.5	TTAGCAGGCGCTTTCAGTATAGATGGCTGGAATTCTCAAGGGTAATCCAGGGGACC ACAACTTTGCTGCCTCAATGGGACAAATGTGTAAACTTTATTGA
**POLR1B**	NM_019014.3	GGAGAACTCGGCCTTAGAATACTTTGGTGAGATGTTAAAGGCTGCTGGCTACAATT TCTATGGCACCGAGAGGTTATATAGTGGCATCAGTGGGCTAGAA
**RPLP0**	NM_001002.3	CGAAATGTTTCATTGTGGGAGCAGACAATGTGGGCTCCAAGCAGATGCAGCAGAT CCGCATGTCCCTTCGCGGGAAGGCTGTGGTGCTGATGGGCAAGAA
**RUNX2**	NM_004348.3	GAAGCCACAGCAGTTCCCCAACTGTTTTGAATTCTAGTGGCAGAATGGATGAATCT GTTTGGCGACCATATTGAAATTCCTCAGCAGTGGCCCAGTGGTA
**SMAD1**	NM_005900.2	CCTTGCATGTACTTGAAGGATGGATGAGTCAGACACGATTGAGAACTGACAAAGG AGCCTTGATAATACTTGACCTCTGTGACCAACTGTTGGATTCAGA
**SMAD2**	NM_001003652.3	GTATGTGTAAACCCTTACCACTATCAGAGAGTTGAGACACCAGTTTTGCCTCCAGT ATTAGTGCCCCGACACACCGAGATCCTAACAGAACTTCCGCCTC
**SMAD3**	NM_005902.3	TTAAAGGACAGTTGAAAAGGGCAAGAGGAAACCAGGGCAGTTCTAGAGGAGTGC TGGTGACTGGATAGCAGTTTTAAGTGGCGTTCACCTAGTCAACACG
**SMAD4**	NM_005359.3	AGGTTGCACATAGGCAAAGGTGTGCAGTTGGAATGTAAAGGTGAAGGTGATGTTT GGGTCAGGTGCCTTAGTGACCACGCGGTCTTTGTACAGAGTTACT
**SMAD5**	NM_005903.5	CCTGATGATCAGATGGGTCAAGATAATTCCCAGCCTATGGATACAAGCAATAATA TGATTCCTCAGATTATGCCCAGTATATCCAGCAGGGATGTTCAGC
**SMAD6**	NR_027654.1	GAATCTCCGCCACCTCCCTACTCTCGGCTGTCTCCTCGCGACGAGTACAAGCCACT GGATCTGTCCGATTCCACATTGTCTTACACTGAAACGGAGGCTA
**SMAD7**	NM_005904.2	AGCAGAAATCCAAGCACCACCAAACACAGTGTATGAAGGGGGGCGGTCATCATTT CACTTGTCAGGAGTGTGTGTGAGTGTGAGTGTGCGGCTGTGTGTG
**SMAD9**	NM_001127217.2	CCAGCACTGGTGCTCGGTCGCCTACTATGAACTGAACAACCGAGTTGGGGAGACA TTCCAGGCTTCCTCCCGAAGTGTGCTCATAGATGGGTTCACCGAC
**SOX9**	NM_000346.2	CAGTGGCCAGGCCAACCTTGGCTAAATGGAGCAGCGAAATCAACGAGAAACTGG ACTTTTTAAACCCTCTTCAGAGCAAGCGTGGAGGATGATGGAGAAT
**SPP1**	NM_000582.2	CGCCTTCTGATTGGGACAGCCGTGGGAAGGACAGTTATGAAACGAGTCAGCTGGA TGACCAGAGTGCTGAAACCCACAGCCACAAGCAGTCCAGATTATA
**TGFB1**	NM_000660.3	TATATGTTCTTCAACACATCAGAGCTCCGAGAAGCGGTACCTGAACCCGTGTTGCT CTCCCGGGCAGAGCTGCGTCTGCTGAGGCTCAAGTTAAAAGTGG
**TGFB2**	NM_003238.2	AAGCCAGAGTGCCTGAACAACGGATTGAGCTATATCAGATTCTCAAGTCCAAAGA TTTAACATCTCCAACCCAGCGCTACATCGACAGCAAAGTTGTGAA
**TGFB3**	NM_003239.2	AGTGCAGTGAGTTCATGCACCTTCTTGCCAAGCCTCAGTCTTTGGGATCTGGGGAG GCCGCCTGGTTTTCCTCCCTCCTTCTGCACGTCTGCTGGGGTCT
**TGFBR1**	NM_004612.2	GAATCCTTCAAACGTGCTGACATCTATGCAATGGGCTTAGTATTCTGGGAAATTGC TCGACGATGTTCCATTGGTGGAATTCATGAAGATTACCAACTGC
**TGFBR2**	NM_001024847.1	ATTTGGAGAATGTTGAGTCCTTCAAGCAGACCGATGTCTACTCCATGGCTCTGGTG CTCTGGGAAATGACATCTCGCTGTAATGCAGTGGGAGAAGTAAA
**VEGFA**	NM_001025366.1	GAGTCCAACATCACCATGCAGATTATGCGGATCAAACCTCACCAAGGCCAGCACA TAGGAGAGATGAGCTTCCTACAGCACAACAAATGTGAATGCAGAC
**VEGFB**	NM_003377.3	TGCCGGAAGCTGCGAAGGTGACACATGGCTTTTCAGACTCAGCAGGGTGACTTGC CTCAGAGGCTATATCCCAGTGGGGGAACAAAGAGGAGCCTGGTAA
**VEGFC**	NM_005429.2	GGCGAGGCCACGGCTTATGCAAGCAAAGATCTGGAGGAGCAGTTACGGTCTGTGT CCAGTGTAGATGAACTCATGACTGTACTCTACCCAGAATATTGGA

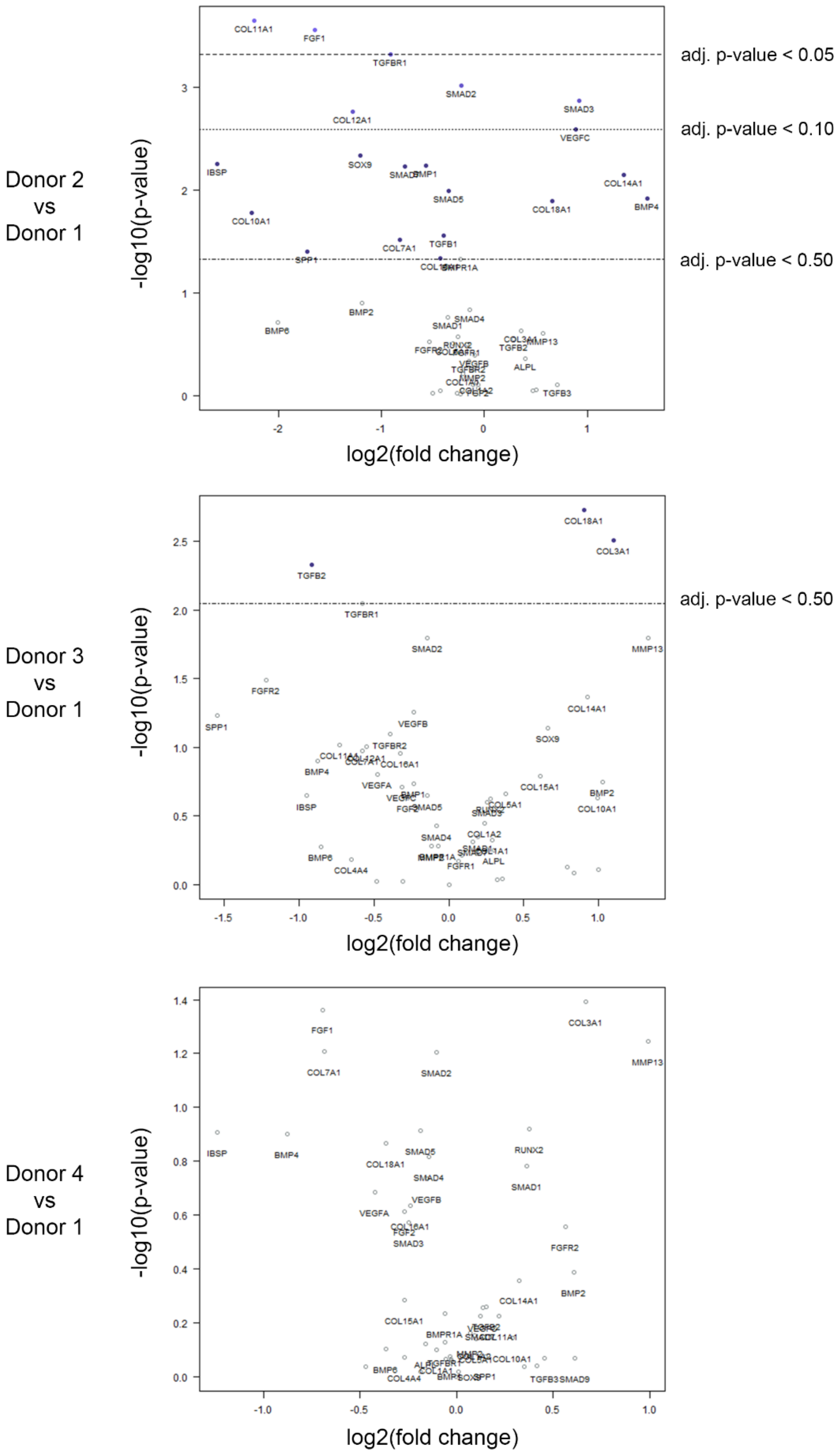

Donor 2
vs
Donor 1
-log10(p-value)
log2(fold change)
adj. p-value < 0.05
adj. p-value < 0.10
adj. p-value < 0.50
COL11A1
FGF1
TGFBR1
SMAD2
SMAD3
COL12A1
VEGFC
IBSP
SOX9
COL14A1
SMAD5
COL18A1
BMP4
COL10A1
COL7A1
TGFB1
SPP1
BMP2
SMAD4
SMAD1
BMP6
ALPL
TGFB3
Donor 3
vs
Donor 1
COL18A1
COL3A1
TGFB2
TGFBR1
SMAD2
MMP13
FGFR2
COL14A1
SPP1
VEGFB
SOX9
TGFBR2
COL16A1
BMP4
VEGFA
COL15A1
BMP2
IBSP
COL10A1
SMAD4
COL1A2
BMP6
COL4A4
FGFR1
ALPL
Donor 4
vs
Donor 1
COL3A1
FGF1
COL7A1
SMAD2
MMP13
IBSP
BMP4
SMAD5
RUNX2
COL18A1
SMAD4
SMAD1
VEGFB
VEGFA
FGFR2
SMAD3
COL14A1
BMP2
COL15A1
BMPR1A
COL10A1
BMP6
COL4A4
SPP1
TGFB3
SMAD9

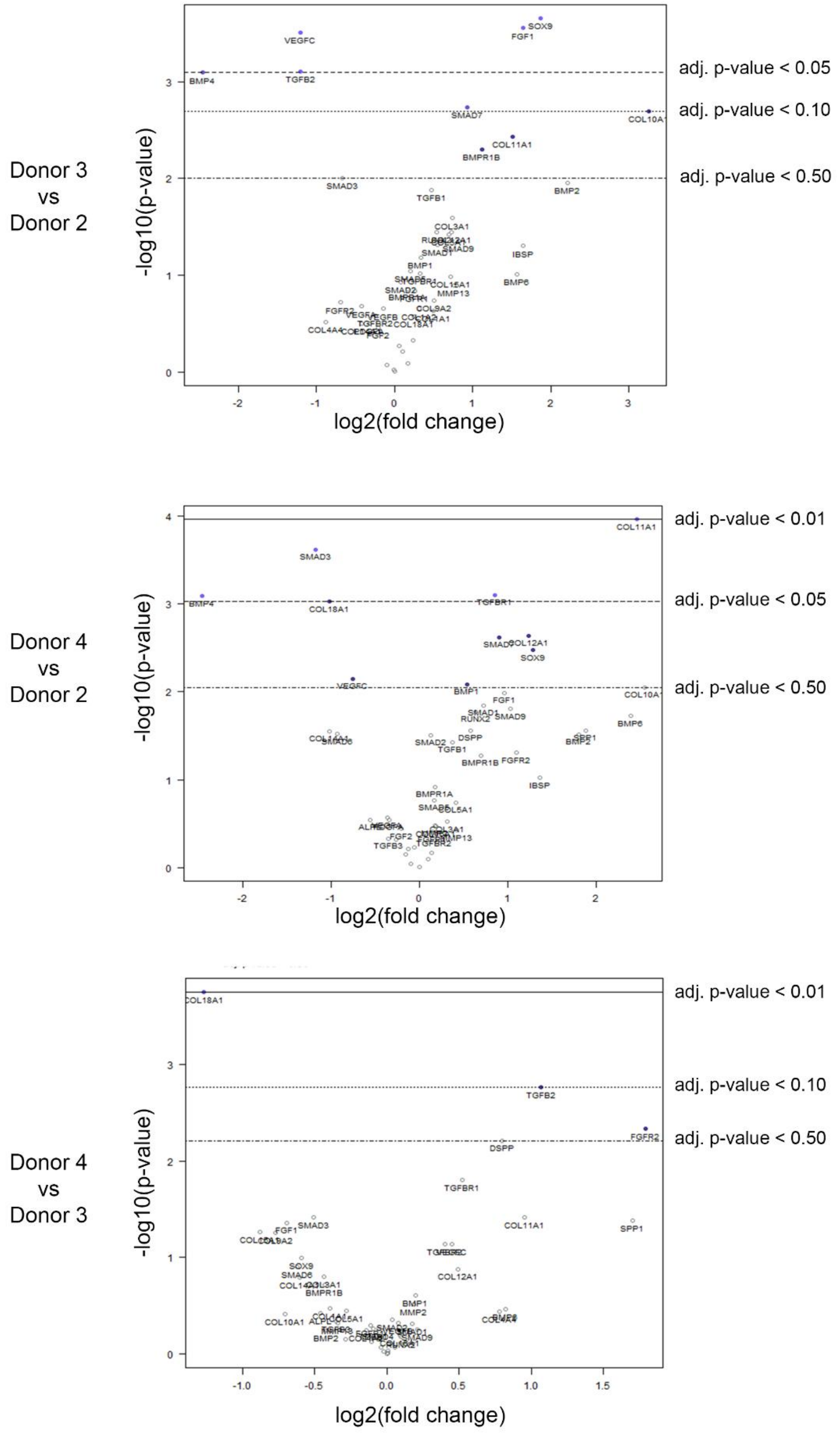

Figure S1: Multivariate analysis of donor variability. Multivariate analysis of the sixty-six tested genes displayed as a volcano plot (created with nSolver® software) with the four donors set as covariate. The plot shows each gene's -$\log_{10}$(p-value) and $\log_2$ fold change with the differentiation set as covariate. Highly statistically significant genes fall at the top of the plot above the indicated p-

value lines, and highly differentially expressed genes fall to either side (right = upregulated, left = downregulated). The comparisons between the donors are shown (Donor 2 vs 1, Donor 3 vs 1, Donor 4 vs 1, Donor 3 vs 2, Donor 4 vs 2, and Donor 4 vs 3). Annotated lines indicate the significance levels.
